# Microbial Diversity of Chronic Wound and Successful Management of Traditional Chinese Medicine

**DOI:** 10.1155/2018/9463295

**Published:** 2018-07-09

**Authors:** Minfeng Wu, Yan Li, Dongjie Guo, Gang Kui, Bin Li, Yu Deng, Fulun Li

**Affiliations:** ^1^Department of Dermatology, Yueyang Hospital of Integrated Traditional Chinese and Western Medicine Affiliated to Shanghai University of Traditional Chinese Medicine, Shanghai 200437, China; ^2^Department of Traditional Chinese Medicine, The Ninth People's Hospital Affiliated to Shanghai Jiaotong University, Shanghai 200011, China; ^3^School of Medicine, Chengdu University, Chengdu 610106, China; ^4^Department of Dermatology, The Seventh People's Hospital of Integrated Traditional Chinese and Western Medicine Affiliated to Shanghai University of Traditional Chinese Medicine, Shanghai 200137, China

## Abstract

Chronic ulcer, including diabetic ulcer, varicose ulcer, and pressure ulcer, negatively affects patients' quality of life. As microbiology plays an important role in the mechanism of pathology for chronic wound healing, this study concentrates on microecology environment of the wound and how Traditional Chinese Medicine (TCM) regulates wound bacteria.* Method*. The study took wound samples from 35 patients and analyzed bacteria variation before and after TCM treatment by 16s rRNA sequencing. All samples were evaluated from aspects of *α*-diversity, *β*-diversity, and Simpson's Diversity index.* Result*. After total DNA extraction, PCR, and 16S rRNA sequencing of wound bacteria from 35 individuals, it was discovered that younger patients with shorter course of disease have a higher microbial diversity and were easier to recover from ulcers. Additionally, gender also played a vital role in wound healing, and a significant microbial diversity existed between male and female patients.* Conclusion*. Patients with chronic ulcers achieved a positive effect after TCM treatment (skin-producing ointment). Mechanistically, TCM helped promote wound healing by regulating the wound microbiota.

## 1. Introduction

Chronic ulcer is a common infectious cutaneous disease, which is characterized by long-term, nonhealing wound, and local bacterial infections [1, 2]. In the US, cutaneous ulcer costs the economy over 25 billion dollars per year for treatment. In the Great Britain, the expenditure on venous ulcer is up to 4 billion pounds per year, with almost 1% of the European population affected by chronic venous ulcers [3]. In China, 1.77% of inpatients are found with nonhealing chronic ulcers [4]. Due to increasing incidences and high therapy costs, chronic ulcers have seriously affected patients' health, mental state, and quality of life. The cause of chronic ulcer is a complex combination of factors, including trauma, dysneuria, circulatory dysfunction (e.g., varicosity), endocrine metabolic disturbances (e.g., diabetes), immune dysfunction (e.g., Behcet's disease), and microbial infection [3, 5, 6]. Moreover, wound healing is also influenced by age, disease course, psychological states, and other unknown factors [7, 8]. These complicated factors result in treatment difficulty and challenges.

At present, therapy for chronic ulcers mainly consists of debridement dressing, He-Ne lasers, antibiotics, immunomodulators, and symptomatic treatments such as controlling diabetes, varicosity, or hypertensive diseases [9, 10]. However, the elderly and people with various internal systemic diseases commonly have poor nutritional status and weak immunity. As recovery of wound can be affected by various factors, these patients might not achieve curative effects after regular treatment of their wounds. Additionally, wounds could also be accompanied by infection, gangrene, and pyogenic osteomyelitis [11, 12]. Recently, Traditional Chinese Medicine (TCM) has become a complementary and alternative medicine worldwide and has gradually been adopted to treat chronic ulcers [13]. In TCM, it is believed that the pathogenesis of chronic ulcer is due to a deficiency of qi and blood, caused by prolonged disease course for healing of the skin wound. Therefore, blood circulation in the leg can be disturbed and necrotic tissues could then generate around the wound, causing occurrence of chronic ulcer. External therapy is one of the most characteristics and preponderant treatments of TCM. In this study, 35 patients were treated with external TCM (skin-producing ointment) made of several herbs, resulting in positive effects.

However, the potential mechanism of how TCM cures chronic ulcer remains elusive. Wound is a microecological environment filled with large amounts of diverse microbes [14, 15]. Metagenomics is a novel method to detect species and diversity of bacteria by high-throughput sequencing technology [16, 17]. In this study, wounds of patients were detected by metagenomic techniques before and after TCM treatment, to explore microbial changes in ulcers. Additionally, patients were also grouped according to their gender, age, and disease course. Comparison of bacteria variation between these groups may provide microbiological information and TCM treatment targets for further study.

## 2. Clinical Case and Treating Efficacy

**Figure 1:**
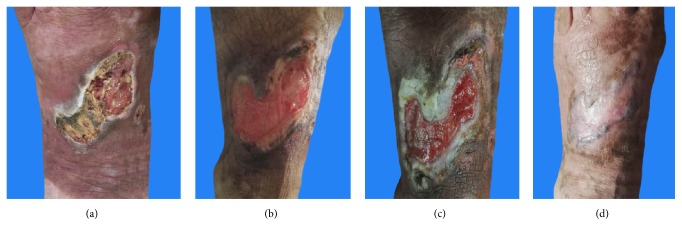
Case presentation of a 69-year-old female patient with chronic ulcer and lesion changes during TCM treatment.

**Figure 2:**
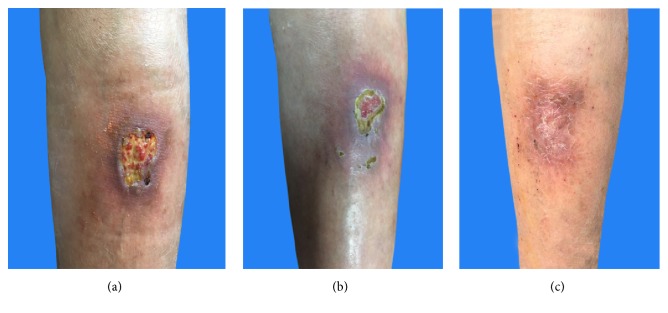
Case presentation of a 56-year-old female patient with chronic ulcer and lesion changes during TCM treatment.

Chronic ulcer is a type of nonhealing wound that has become a heavy economic burden for patients. A total of 35 patients with disease course over 1 month were included into the study and treated with a skin-producing ointment. Hereby, we presented two clinical cases with positive therapeutic effect. One case is a 69-year-old Asian female patient with a chronic ulcer on the left foot for over 2 years and the condition was in the progressive stage (Figure 1(a)). She was treated with sulfadiazine silver ointment and nitrofurazone solution during her disease course, but no obvious effect was achieved. After topical application of skin-producing ointment for 2 weeks, effluvial pus and exudate had been largely eliminated (Figure 1(b)). Through another 2 weeks of treatment, wound proportion decreased upon visual inspection (Figure 1(c)). Finally, the wound completely scabbed after the entire therapy duration of 2 months (Figure 1(d)). The patient was followed up for one year with no recurrence. Another case was a 56-year-old Asian female patient with chronic right leg ulcer for over 10 months. The wound was due to an accidental trauma during a fall. She did not go to hospital for local dressing changes but sterilized wound by 75% ethyl alcohol and mupirocin ointment at home. However, the wound showed no tendency to heal and wound area was continuously enlarging from a small cut(Figure 2(a)). Severe pain (VAS=8/10) also appeared after application of 75% ethyl alcohol over wound. After topical application of skin-producing ointment for one month, an wound area decreased upon visual inspection and pus can be seen (Figure 2(b)). Additionally, the patient only suffered from very limited pain (VAS=2/10) during treatment. The wound completely healed and left with some hyperpigmentation after completion of the two month therapy (Figure 2(c)).

## 3. Materials and Methods

### 3.1. Sample Collection and Patient Information

Patients in this study were recruited from Yueyang Hospital of Integrated Traditional Chinese and Western Medicine Affiliated to Shanghai University of Traditional Chinese Medicine, between September 2016 and July 2017. This study has been approved by the Ethics Committee of Yueyang Hospital of Integrated Traditional Chinese and Western Medicine Affiliated to Shanghai University of Traditional Chinese Medicine, protocol 2016061.

Patient inclusion criteria are as follows: (1) age of 18 to 75 years old; (2) disease course of over 1 month and with no healing tendency after routine dressing therapy; (3) diameter of wound size 2–10 cm; (4) being with agreement to attend clinical trial observation and follow-up on time; (5) signature on the informed consent form.

Patient exclusion criteria are as follows: (1) having received steroids or immunosuppressors within 2 weeks; (2) being allergic to any constituents of studied medicine; (3) being pregnant or breastfeeding or becoming pregnant during the treatment course; (4) having cancer-related, tubercular, and radiation ulcer; (5) being accompanied with serious heart, liver, or kidney diseases.

Before sampling, wound edges would cleaned by 0.9% normal saline (NS) first. Then sterile cotton swab was used to slightly press the middle of wound tissues and 2cm around wound, thereby getting pus from ulcer. All the samples were stored in cubes containing 0.9% NS at the temperature of -20°C and sent out for inspection. Samples were taken from every patient during the first visit and at the end of treatment course (6 weeks of skin-producing ointment therapy). After cultivation and identification, pus samples of 35 patients all met microbial standards and had enough testing volume of over 1ml.

Skin-producing ointment is prepared by the Ninth People's Hospital Affiliated to Shanghai Jiaotong University, which contains Elephas maximus L., Lithospermum erythrorhizon Sieb. et Zucc, Rehmannia glutinosa Libosch, Cortex Lycii, Angelica sinensis, Rheum palmatum L, mercurous chloride, and Glycyrrhiza uralensis Fisch. This ointment was clinically applied to treat chronic nonhealing ulcer as it has the specific function of removing slough, regenerating granulation tissues, and supplementing potential ulcer cavity and alleviating pain.

### 3.2. Total DNA Extraction, PCR, and 16S rRNA Sequencing

**Figure 3:**
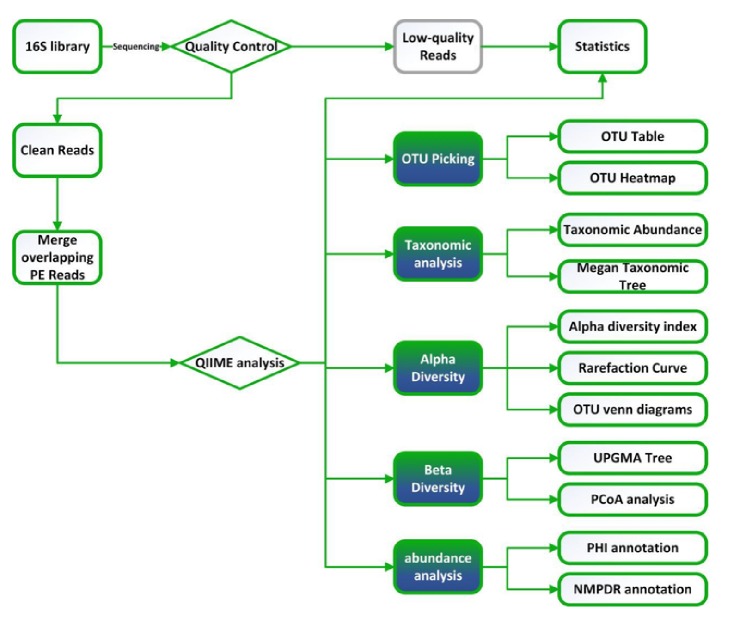
Flow diagram of biological information analysis.

Bacterial DNA was extracted directly from wound samples using the QIAamp Fast DNA Stool Mini Kit following the manufacturer's protocol (Figure 3). Genomic DNA was quantified using the Qubit® assay kit and NanoDrop Spectrophotometer and stored at −20°C.

The 16S ribosomal RNA (rRNA) amplicon region was amplified using barcoded ‘universal' bacterial primer 27F (5′-AGAGTTTGAT CCTGGCTCAG-3′) and 515R (5′-ATTACCGCGGCTGCTGG-3′); each primer had different barcodes and the same adaptors. The barcoded primers allow pooling of multiple PCR amplicons in a single sequencing run. PCR was carried out using the reaction conditions as follows: 50°C 2min, 95°C for 3min (denaturing) followed by amplification for 30 cycles at 95°C for 20s, 58°C for 30 s and 72°Cfor 2min, and a final extension of 72°C for 2 min. PCR amplicons were purified and sequenced on the Illumina MiSeq platform [18].

### 3.3. Preprocess

FASTQC (http://www.bioinformatics.babraham.ac.uk/projects/fastqc) is used for the quality control to check on the quality of raw data. Because the data is generated by Illumina, the universal adaptor from the raw data is removed by CUTADAPT (http://code.google.com/p/cutadapt/). Pair-end reads are joined together using FLASH (https://sourceforge.net/projects/flashpage/files/). The Fastq sequence was made compatible with “split_libraries_fastq.py” by cutting the barcodes from the Fastq sequence. Reads are demultiplexed using the Quantitative Insights into Microbial Ecology (QIIME v.1.8.0) pipeline [19]. First, use “split_libraries_fastq.py” which performs demultiplexing of Fastq sequence data where barcodes and sequences are contained in two separate Fastq files above (common on Illumina runs) into a fasta file which contains the sequence of 16S rRNA and a fasta file of the quality of the sequence file. Chimera sequences in the data are removed using “identify_chimeric_seqs.py” which identify chimeric sequences in input FASTA file with usearch61 algorithm to identify the chimera, and using “filter_fasta.py” module, the chimeric sequences is removed.

### 3.4. Clean Data Analysis

Sequences were grouped into operational taxonomic units (OTUs) at 97% sequence similarity using the Greengenes reference database. OTUs that did not cluster with known taxa at 97% identity or higher in the database were clustered de novo and not de novo (UCLUST) (Edgar, 2010). Representative sequences for each OTU were then aligned using PyNast [20], and taxonomy was assigned using the RDP classifier (Version 2.2) [21]. A phylogenetic tree was built using FastTree. “split_otu_table.py” splits a biom table base on the group which is contained in mapping file. Venn diagram was generated with R by the otu table and group. Taxonomic information is necessary, and “summarize_taxa.py” provides the abundance information which can be visualized by histogram [22]. Alpha diversity and beta diversity are important for the analysis. Alpha diversity showed in Table 1 is analyzed by the module of “alpha_diversity.py” and generated alpha rarefaction plots with five indexes, Shannon, Chao1, observed_species, goods_coverage, and Simpson, to show the diversity of species. Beta diversity is analyzed by using “beta_diversity_through_plots.py” to generate two 3D and two 2D principal coordinate analysis (PCoA) plots, weight, and unweight UniFrac [23]. To directly measure the robustness of individual UPGMA clusters and clusters in PCoA plots, we performed jackknifing (repeatedly resampling a subset of the available data from each sample).

## 4. Results and Discussion

### 4.1. *α*-Diversity Index Statistics

**Figure 4:**
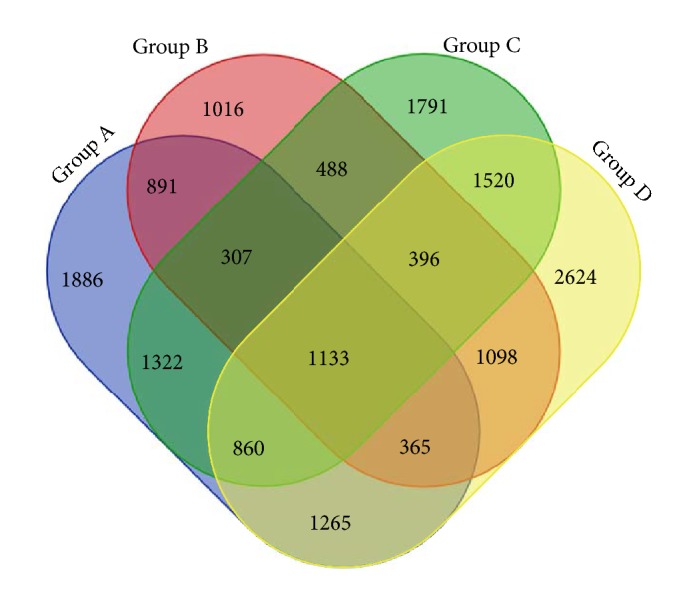
Venn diagram: number of common and specific wound microbes before and after TCM treatment.

In the field of ecology, *α*-diversity index represents the biological diversity of a specific ecosystem. As single diversity index has bias of estimator, several related diversity indexes are usually calculated at the same time to evaluate the result objectively. In this study, Shannon, Chao1, observed species, goods coverage, and Simpson index were applied to analyze biological diversity from the all-sided aspects of community diversity, bacteria abundance, species number under visual inspection, sequencing depth, and random selection probability with the assistance of QIIME v1.8.0 software. The biological diversity is higher as the index gets closer to 1. As shown in the Figures 1 and 4, Simpson indexes of four groups are all over 0.96 and goods coverage indexes are all over 0.98, representing a high biological abundance.

In the Venn diagram, Groups A and B represent the microbe samples of skin around ulcer wound before and after treatment, while Groups C and D represent for microbe samples of ulcer wound before and after TCM treatment. It can be discovered from the diagram that Groups A, B, C, and D contain, respectively, 8029, 5694, 7817, and 9261 OUT (Operational Taxonomic Units), symbols set for sorting out the same kind of species or strains of microbes. By contrast, Group D has the most abundant microbes and Group B has the least, which indicates that microbial diversity of lesions within wound is less multiple than that of skin around ulcer. In addition, before treatment the wound contains 7817 OTU and after treatment the OTU of wound has increased up to 9261 OTU. Thus, the TCM treatment (skin-producing ointment) upregulated wound microbial diversity to make the microecological environment of wound more similar to normal skin around the wound. The process of changing the microbiome within the wound might be a potential mechanism of action for TCM in treatment of chronic ulcer.

### 4.2. Rarefaction Curve Analysis

**Figure 5:**
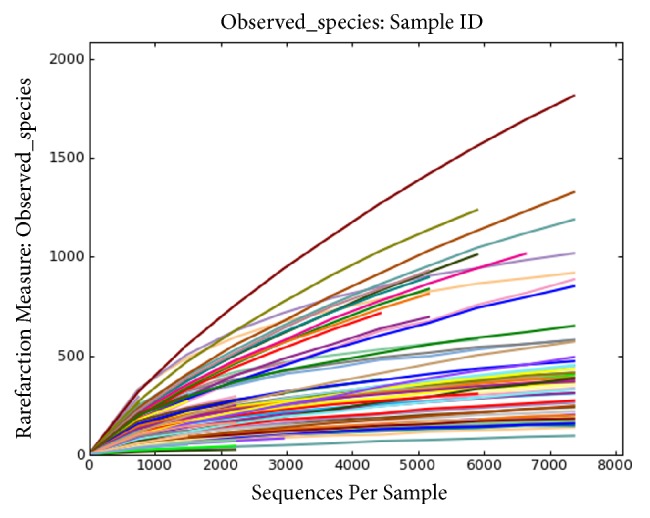
Diversity index “observed species” of species rarefaction curve.

This study includes 35 chronic ulcer patients and plots the result by QIIME v1.8.0 software. We adopted random sampling method to select sequence number and to calculate species rarefaction curve (an indicator to reflect data sequencing depth). The steep slope of Figure 5 represents that more samples are needed to verify data robustness reasonableness. The flat part of graph shows that species rarefaction curves tend to be flat with continuous increase of sequencing depth, which illustrates that sample amount has already reached saturation point. In this graph, rarefaction curve tends to be flat when sequencing depth reaches to 7000 reads per sample and accounts for enough samples of the experiment.

### 4.3. *β*-Diversity and Microbial Difference Analysis

**Figure 6:**
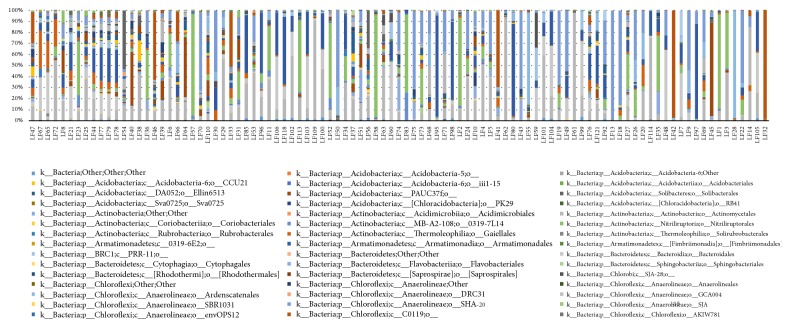
Microbial diversity and bacteria proportion in wound and skin around wound before and after TCM treatment.

In this study, 35 patients were included whose fester was extracted from the wound and detected by 16s rRNA high-throughput sequencing. Samples were taken from wound and normal skin around wound both before and after TCM treatment. As is shown in the diagram, samples of every individuals varied significantly due to personal difference and changes of patients' microbial wound could be found with the progress of therapy. The microbial diversity and bacteria proportion can be seem from Figure 6. The microbes were observed and evaluated at the biological classification level of order. In general, proteobacteria, firmicutes, bacteroidetes, and actinobacteria were the top four dominant microbes in patients' samples. Both Gram-negative and Gram-positive bacteria could be identified in the samples. In order to further understand the wound microbial diversity, we divided these samples into several groups according to different factors. The data can help reveal clinical characteristics and explain the potential mechanism behind the clinical manifestation, which may provide guidance for therapy.

**Figure 7:**
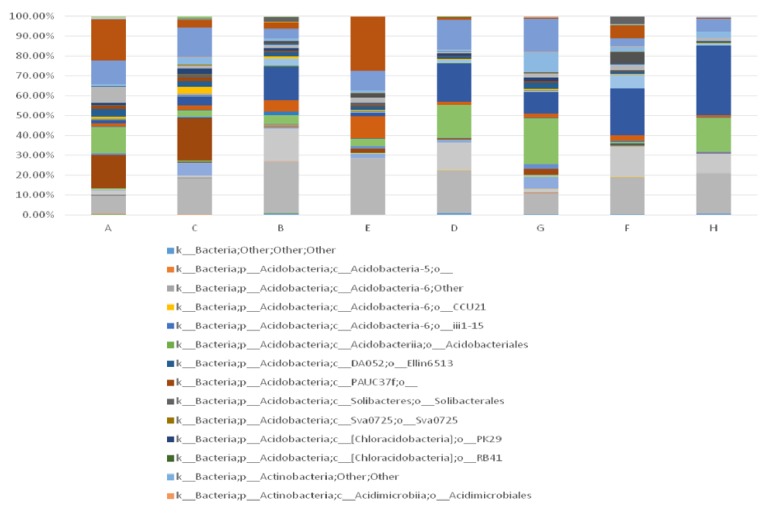
Microbial alteration of wound as well as skin around wound before and after TCM treatment, and its relationship with gender. (Note: A, B: lesions around wound before treatment (male and female); C, D: lesions around wound after treatment (male and female); E, F: lesions within wound before treatment (male and female); G, H: lesions within wound after treatment (male and female). All the bacteria were observed at the biological level of order.)

It can be found from Figure 7 that, besides TCM treatment, gender also affects microbial alteration of wound. The difference and alteration are mainly reflected in the types and structural proportion of the bacteria. Bacteria of lesions around the wound did not change significantly before and after TCM treatment. However, lesions within wound showed a distinct microbial alteration during therapy. We observed that this alteration is dependent on the gender of the patient, which warrants further study. Before treatment, 49 and 59 species of bacteria were detected from men's and women's wound, respectively. The top 3 dominant wound bacteria of women were clostridiales (23.45%), actinomycetales (18.8%) and bacteroidales (14.4%), while the top 3 dominant wound bacteria of men were actinomycetales (20.95%), xanthomonadales (18.28%), and lactobacillales (14.36%). After treatment, it is interesting that men's wound (78 species) showed more diverse bacterial types than women's wound (59 species). At this time, the top three dominant wound bacteria of male were bacillales (22.84%), pseudomonadales (16.67%), and clostridiales (10.87%), while the top three dominant wound bacteria of female were clostridiales (35.25%), actinomycetales (20.16%), and bacillales (16.83%). As a result, it can be concluded that dominant bacteria and bacterial species in wound of female patients changed little after TCM treatment. For male patients, the top three dominant bacteria had varied, and bacterial species had greatly increased by 29 species of bacteria, indicating an obvious increase of biological diversity after TCM treatment. However, the potential mechanism for gender to affect the development process of chronic ulcer requires further research and greater clinical samples.

**Figure 8:**
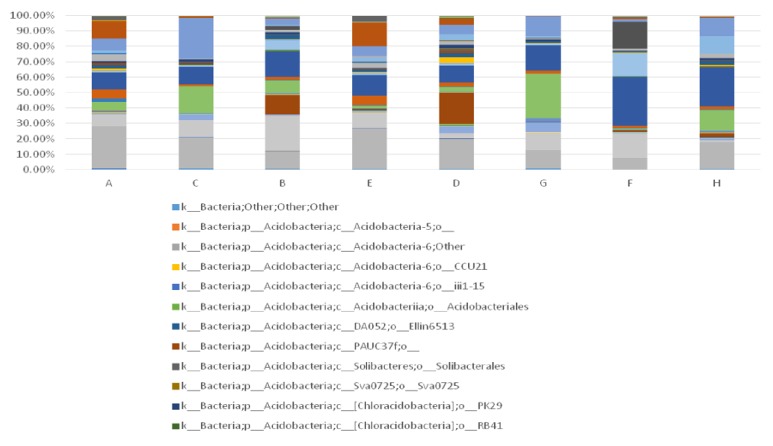
Microbial alteration of wound as well as skin around wound before and after TCM treatment and its relationship with ages. (Notes: A, B: lesions around wound before treatment (over 65 years old and under 65 years old); C, D: lesions around wound after treatment (over 65 and under 65); E, F: lesions within wound before treatment (over 65 and under 65); G, H: lesions within wound after treatment (over 65 and under 65). All the bacteria were observed at the biological level of order.)

As chronic ulcer is more prevalent in aged population, it is speculated that age may play an important role in this disease. In total, 35 patients were included into the study, divided into two groups according to age. After statistics and grouping, 16 patients were under 65 years old and 19 patients were over 65. The youngest was 54 years old, while the oldest was 75 years old. Before treatment, 47 and 42 species of bacteria were detected from the wound of patients below and over 65 years old, respectively. In contrast, wound of patients below 65 contained more bacteria types than patients over 65. The top 3 dominant wound bacteria of patients below 65 were clostridiales (31.96%), campylobacterales (16.97%), and bacteroidales (15.94%), while those of patients over 65 were actinomycetales (26.32%), xanthomonadales (15.32%), and clostridiales (13.42%). It was significant that after treatment wound of patients below 65 still contained more bacteria types than patients over 65. The top three dominant wound bacteria of patients below 65 were clostridiales (25.66%), actinomycetales (17.06%), and bacillales (13.83%), while those of patients over 65 were bacillales (28.95%), clostridiales (16.52%), and pseudomonadales (12.81%). Through comparison, bacteria species for patients below 65 greatly increased from 47 to 83 with TCM therapy, while bacteria species for patients over 65 increased from 42 to 57. Either patients below or over 65 show a distinct microbial alteration after TCM therapy, and both dominant bacteria types and total structural proportion varied with treatment progress. For both patients below and after 65, the most dominant wound bacteria were replaced except clostridiales. In addition, it can be found from the graph that degrees of variation were more obvious for patients below 65 than those over 65 (Figure 8).

It can then be speculated that age could be a vital factor which affects healing of chronic ulcer. It is likely that better nutrition and therefore better blood circulation could result in a more abundant biological diversity in younger populations than the aged population. Furthermore, they are also more sensitive to TCM treatment than the aged as degree of variation in patients below 65 was remarkably higher than those over 65 after TCM therapy. So, it can also be concluded that wounds in younger population have richer bacteria species present and tend to heal more quickly than the aged.

**Figure 9:**
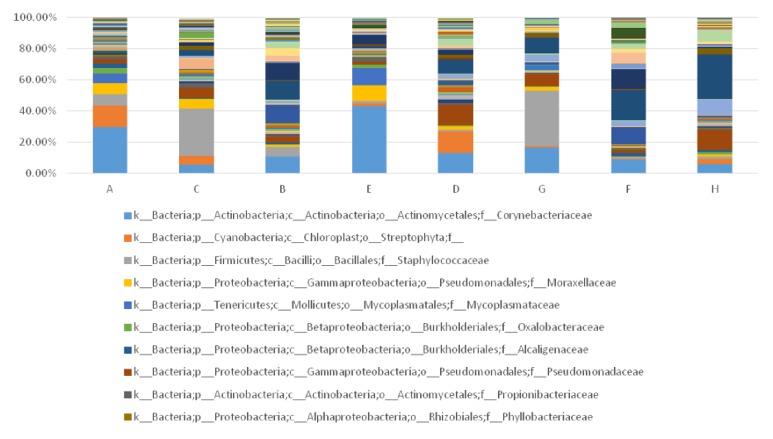
Microbial alteration of wound as well as skin around wound before and after TCM treatment and its relationship with disease courses. (Notes: A, B: lesions around wound before treatment (disease course over and below half year); C, D: lesions around wound after treatment (disease course over and below half year); E, F: lesions within wound before treatment (disease course over and below half year); G, H: lesions within wound after treatment (disease course over and below half year). All the bacteria were observed at the biological level of order.)

Long disease courses have been associated with difficulties in recovery. As patients of different disease course have disparate onset time and responses to therapy, there could be a relationship between disease course and outcome. Therefore, 35 patients were divided into two groups according to their disease course (disease course over 6 months and below 6 months). In total, 17 patients have disease courses that were over 6 months and 18 were below 6 months. In addition, the shortest was 3 months, while the longest was 5 years. Before treatment, 51 and 56 species of bacteria were detected from the wound of patients with disease courses more than 6 months and less than 6 months, respectively. Actinomycetales, pseudomonadales, and mycoplasmatales were the top three dominant wound bacteria of patients with longer course, while clostridiales, bacteroidales, and xanthomonadales were the top three dominant wound bacteria of patients with shorter course. After treatment, 61 and 81 species of bacteria were detected from the wound of patients with disease courses more than 6 months and less than 6 months, respectively. Bacillales, actinomycetales, and clostridiales were the top three dominant wound bacteria of patients with longer courses, while clostridiales, pseudomonadales, and enterobacteriales were the top three dominant wound bacteria of with shorter courses. Through comparison, it can be found that bacteria species of patients with shorter courses increased by 25 (from 56 to 81) after TCM treatment, while bacteria species of patients with longer courses only increased by 10 (from 51 to 61). Three dominant wound bacteria of the shorter-course group were entirely replaced with another three new bacteria, while the dominant wound bacteria of the longer-course group were replaced with two new bacteria. In conclusion, the shorter-course group showed a more abundant microbial diversity, higher degree of variation, and more obvious changes of dominant bacteria in contrast with the longer-course group. As is shown in Figure 9, wound with shorter disease courses contains more species of bacteria either before (56:51) or after (81:61) TCM treatment. However, the reason why the shorter-course group exhibited a higher microbial diversity and achieved a better outcome demands larger samples to explore.

### 4.4. *β*-Diversity Analysis

**Figure 10:**
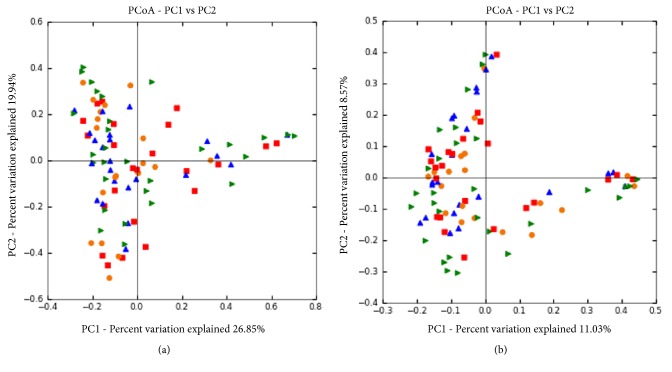
PCoA results based on different algorithms: (a) Weighted UniFrac (Consider sequencing amounts when analyzing); (b) Unweighted UniFrac.

PCoA (principal coordinates analysis) is a method to discover differences between individuals or groups by ranking eigenvalues and eigenvectors and selecting out the top-ranking ones. UniFrac is an index to calculate distance between microbiome evolution sequence and reflect whether there is significant difference within environmental sample. Every dot in the graph represents an independent sample from the patient's lesion. As dots with four colors represent four groups (wound and normal skin around wound before and after TCM treatment), it can be found from the graph that four groups of dots are equally distributed (Figure 10). Thus, difference between four groups of our study has statistical significance.

## 5. Conclusions

Chronic ulcer is a clinically common cutaneous disease. In the US, it affects 6 million chronic ulcer patients and its treatment costs US$ 2.5 billion. Moreover, Western countries spend 1% of total healthcare budget on ulcer treatment [24, 25]. The delayed healing wound remains a tough problem, as microbiome infection, dysneuria, immune deficiency, and blood circulation disorder result in complex pathogenesis and difficult therapies [26]. The delayed wound healing has already become a serious social-economic burden. Recently, management of chronic ulcer mainly includes debridement (mupirocin ointment, nitrofurazone solution, and rb-bFGF spray), physiotherapy (He-Ne lasers, infrared ray, and ultrashort wave), surgery, symptomatic treatment (anti-infection, anti-inflammation, pain easing, etc.), managing multifactorial diseases (diabetes, varicosity, hypertensive disease, etc.), and TCM therapy [24, 27, 28]. Clinically, progress of chronic ulcer can be affected by various factors, such as microbial infection, nutrition condition, and mental state, which may lead to poor curative effect and prolonged therapy course [29]. An effective treatment has remained a challenging problem. TCM has therapeutic effects and has been adopted to treat chronic ulcers since ancient times in China. Particularly, external application of herbs over wound can achieve the most obvious effect [13]. However, it remains unclear whether TCM exerts its effect through eliminating bacteria or balancing microbiome proportion of wound. Other questions, such as how TCM could help improve tissue granulation to increase healing and other potential mechanism of TCM, still demand further research.

Metagenomics is a novel method to explore population structure, diversity, and evolutionary relation of microbiome through high-throughput sequencing [30]. It could find relationships between bacterial function, structure, and wound environment without cultured microbiome. The research progress of metagenomics mainly contains three steps: extraction of metagenome, data library construction, and screening of target genes [31, 32]. After the complete procedure, abundant genetic and microbial information of microorganism can be obtained for further research.

This study used metagenomics to analyze microbial diversity of chronic wound, to compare microbial population of normal skin with wound, and to discover functional difference among dominant species, shedding light on how microbiome might affect chronic wound healing. We compare microbiome of normal skin with wound before and after treatment to explore whether skin-producing ointment regulates wound microecological environment. As is shown in results, skin-producing ointment can improve wound healing through increasing and decreasing proportion of specific bacteria. Although TCM cannot deactivate bacteria directly, it can regulate ratio of microbes and keep a certain amount of fester in order to provide a favorable wound microenvironment. In theory of TCM, skin-producing ointment has the function of reinforcing deficiency, removing blood stasis, and promoting tissue granulation. Either from the aspect of Western medicine (metagenomics) of TCM, skin-producing ointment can provide nutrition for generating granulation and improve blood circulation around wound.

In this study, we included 35 individuals and detected V1-V3 variable region 16s rDNA sequence of wound by high-throughput sequencing technique. The results were evaluated from the aspects of *α*-diversity, rarefaction curve, *β*-diversity, and PCoA analysis. Firstly, we observed *α*-diversity by recording Shannon, Chao1, observed species, goods coverage, and Simpson index. It was found that these Simpson indexes were all over 0.96, which indicates a high biological abundance. Secondly, we evaluated species rarefaction curve by QIIME v1.8.0 software. The graph indicates that rarefaction curve tends to be flat when sequencing depth was up to 7000 reads per sample, which indicates enough samples for the assay. Next, we observed *β*-diversity by comparing wound microbial variation before and after TCM treatment in order to acquire species diversity and function. In this study, we mainly focused on the role of age, gender and disease course on chronic ulcer microecological environment. With regard to gender, men and women displayed microbial diversity both before and after TCM treatment. Before treatment, women's wound showed higher bacterial diversity than men. Actinomycetales, xanthomonadales, and lactobacillales were the top three dominant wound bacteria for male patients, while clostridiales, actinomycetales, and bacteroidales were the top three dominant wound bacteria for female patients. After treatment, it is interesting that men's wound showed higher bacterial diversity than women's wound. Bacillales, pseudomonadales, and clostridiales were the top three dominant wound bacteria for male patients, while clostridiales, actinomycetales, and bacillales were the top three dominant wound bacteria for female patients. It can be found that dominant bacteria and bacteria types of women's wound changed little after treatment. But for men dominant bacteria have largely changed and bacteria types have obviously increased after TCM treatment. With regard to age, it was reported that chronic ulcer is prevalent in people over 65 years old [26]. In our study, patients over and below 65 years old showed different microbial diversity before and after TCM treatment. Clostridiales, campylobacterales, and bacteroidales were the top three dominant wound bacteria for patients below 65, while actinomycetales, xanthomonadales, and clostridiales were the top three dominant wound bacteria for patients over 65. After treatment, 83 and 57 species of bacteria were detected from the wound for patients below and over 65 years old, respectively. Clostridiales, actinomycetales, and bacillales were the top three dominant wound bacteria for patients below 65, while bacillales, clostridiales, and pseudomonadales were the top three dominant wound bacteria for patients over 65. The results indicated that wound of younger patients exhibited a higher microbial diversity than older patients. The reason why it takes shorter time for younger patients to recover may be related to abundant biological diversity, stable proportion structure, and better systemic nutrition state. These factors all lead to accelerated wound healing. With regard to disease course, it can be found from the sequencing data that wound of patients with shorter course contained larger amounts of bacteria than both before and after TCM treatment. In addition, patients with shorter course also showed a higher degree of variation and more obvious changes of dominant bacteria after TCM treatment. Finally, we also researched PCoA of our samples to know whether difference between four groups of our study has statistical significance. Since every dot in the graph represents for an independent sample, it can be discovered from the graph that four groups of dots (represented by four groups) are equally distributed.

Skin is the largest organ of human and thus contains many complex microbial environments. Researchers realized that pH and temperature are disparate in different parts of human body. The human body surface has a pH range from 4.2 to 7.9 while the temperature is from 29.5 to 36.6°C. Factors including sex, age, injury, anxiety, drugs, nutrition, UV exposure, and lifestyles may all lead to stimulation of innate immunity, thus regulate microbiome on the skin surface [33]. The microbial environment of cutaneous diseases such as ulcers could be even more complicated and worthy of further study. For ulcer therapy, antibiotics are a commonly used method [34]. Benjamin A. Lipsky et al. conducted randomized, controlled, double-blinded multicenter trial and demonstrated that topical pexiganan had a better effect than oral antibiotic in mildly infected diabetic foot ulcers. A combined therapy of topical pexiganan and applicable wound care can offer a therapeutic method to a broad-spectrum oral antibiotic agent. Moreover, it is also safe and can prevent the appearance of resistant bacteria [35]. Drug resistance may easily occur after long-term application of continued or multiple antibiotics. Antibiotics can provide a favorable effect at the beginning of therapy but complex negative side effects at later stages [36]. Ozlem Kandemir et al. also investigated high risk factors for diabetic foot ulcer with multidrug resistant microorganisms. They found that ulcer infection might deteriorate if antibiotics are not properly selected [37].

Recently, more researchers have realized the importance of microbes in chronic ulcers [38, 39]. Hossien Parsa et al. concentrated on microbiological features and risk factors of ulcers. After observation of 54 patients, they found that P. aeruginosa (35%), S. aureus (19%), and MRSA (6%) were the most three significant bacteria in ulcer wound. Age over 65 years, ulcer size over 2 cm^2^ and HbA1c > 7% were the top 4 risk factors of chronic ulcers [40]. In Germany, microbial detection has been widely adopted in diagnosing chronic wounds, especially in patients with deep ulcer and diabetic foot syndrome. Andreas Schwarzkopf et al. also proposed that it would be better to combine biopsy if swabs failed to show pathogens [41]. Ozer B et al. investigated infections and aerobic bacterial pathogens in diabetic foot ulcer. They found causative pathogens, antimicrobial susceptibility, and lesion severity came up as three important factors of ulcer infections. Additionally, Enterobacteriaceae (36.5%), Pseudomonas aeruginosa (18.9%), Enterococcus spp. (14.9%), and Staphylococcus aureus (10.8%) were the top 4 frequent microbes. Vital factors for delayed wound healing mainly include inappropriate antibiotic treatment, long disease course, and frequent hospital admission [42].

M. Malone et al. described the diabetic ulcer microbiome by next generation DNA sequencing and speculated that duration of ulcer might play a decisive role in ulcer microbiome. They found that diabetic ulcer with a shorter duration has a simpler microbiome composition of pyogenic cocci, but ulcer with a longer duration mainly has a main composition of polymicrobial microbiome [43]. Similarly, Sue E. Gardner et al. discovered that duration and ulcer depth play a vital role in wound microenvironment. Patients with deep ulcers and longer disease duration have a higher microbial diversity and specific pathogens. Anaerobes and Proteobacteria are the dominant bacteria in those patients' wound. However, higher abundance of Staphylococcus is found in patients' with superficial ulcers shorter duration [44].

Researchers from University of Cambridge observed 9 patients with diabetic foot ulcer, where both Gram-positive and Gram-negative organisms could be isolated from wound. Additionally, P. aeruginosa, S. aureus, Escherichia coli, S. epidermidis, and Proteus were the most common bacteria in diabetic foot ulcer [45]. Similar study also discovered that Staphylococcus aureus and Streptococcus agalactiae are prevalent in acute ulcer, while anaerobic organisms and Pseudomonas aeruginosa are prevalent in chronic ulcer [46]. Karen Smith et al. sequenced and compared wound of new and recurrent diabetic foot ulcers. They found that these wounds share Peptoniphilus spp., Anaerococcus spp., and Corynebacterium spp. in common, while Staphylococcus spp. was only specific in new ulcers. Nearly 67 % of OTU's residing in new and recurrent ulcers belonged to Gram-positive organisms. They proved the vital role polymicrobial biofilm and polymicrobial interactions play in ulcer, which may help guide efficient treatment [47]. French researchers found that Staphylococcus aureus is a common colonizer both in normal human skin and in diabetic foot infections. Among various bacteria, Staphylococcus aureus took the largest proportion of diabetic ulcer [48]. Study from Morocco also revealed the microbiology of diabetic foot ulcer and found 43% of ulcer infection was related to Gram-negative bacilli though S. aureus was the most prevalent bacteria [49]. Based on previous study, researchers from the US compared microecological environment between diabetic (30 patients) and nondiabetic ulcer (30 patients) through high-throughput 16S rRNA sequencing technique. By contrast, they found that diabetic ulcer showed increased populations of S. aureus and higher bacterial diversity, which might potentially affect wound infection [50].

Infection is considered to be the key factor of chronic ulcer [51]. Benjamin A. Lipsky et al. did the research and suggested that aerobic Gram-positive cocci are the most important bacteria of common chronic ulcer. However, Gram-negative bacilli and anaerobes both participate in the polymicrobial infection [52]. It frequently occurs in complex and previously treated wounds. They demonstrated that chronic lesions in patients who have received previous antibiotic treatment are usually full of microbial diversity. Anaerobes are the main flora in wounds with necrotic tissue and Pseudomonas can usually be isolated from soaked foot ulcer [53]. Recently, more researchers are trying to develop novel therapeutic method to promote wound chronic wound healing. University of Manchester from the UK has developed a collagen wound model and focused on its role of simulating the activity and distribution of antimicrobials in soft tissues of diabetic ulcer. During the experiment, they found that the collagen wound model can help biofilm growth and absorbing serum to simulate the microbial diversity of the microecological environment of chronic ulcer. This model can provide a Pseudomonas aeruginosa biofilm, which offers an efficient alternative way to infection management and wound healing. This work created a new biological material to promote the movement of antimicrobials. As a result, they suggested a combined therapy of antibiotics and calcium sulfate beads for treating delayed healing ulcer [54]. Negin Soroush et al. explored association between vitamin D receptor gene FokI and oxidative stress in ulcers. They discovered that ulcer patients usually present with higher levels of TBARS and FokI gene polymorphism [55]. However, the exact mechanism of how FokI affects oxidative stress and microecological environment requires further research.

Management of chronic ulcer remains a big challenge. Many patients have achieved favorable effect after TCM therapy in our clinical study. However, limitations and defects also exist in this study. Although our research has already revealed changes in types and proportion of the microbiome, it remains unclear which microbiome results in or could aggravate ulcer. Within the entire wound microecological environment, minimizing species of bacteria that could contribute to infection while maximizing the species of bacteria that could help improve healing is a topic worth exploring. The mechanism how TCM regulates microbial balance and promotes wound recovery is worthy further study. In subsequent study, larger number of samples will be needed to reveal the therapeutic effect and microbial diversity of TCM therapy.

## Figures and Tables

**Table 1:** Summary of *α*-diversity index. (Notes: Groups A and B represent microbe samples of skin around ulcer wound before and after treatment, while Groups C and D represent for microbe samples of ulcer wound before and after TCM treatment.)

Group	Shannon	Chao1	Observed species	Goods coverage	Simpson
A	8.379543858	10459.15393	8029	0.983027125	0.982471386
B	8.179379127	10467.99452	7817	0.982466815	0.980780295
C	7.33404199	8263.451099	5694	0.983198157	0.970101782
D	7.554650169	11153.77176	9261	0.987819343	0.968915978

## Data Availability

All of the data used to support the findings of this study are available from the corresponding author upon request.
